# Supplementary material for: Labelizer: systematic selection of protein residues for covalent fluorophore labeling
Source: Nat Commun. 2025 May 4;16:4147. doi: 10.1038/s41467-025-58602-y (PMC12049551; doi:10.1038/s41467-025-58602-y)
Supplement: Supplementary file 1 — Supplementary Information [file 41467_2025_58602_MOESM1_ESM.pdf]

Supplementary Information for

Labelizer: systematic selection of protein residues for covalent fluorophore labelling

Christian Gebhardt[1,*], Pascal Bawidamann[1], Anna-Katharina Spring[1,2], Robin Schenk[3], Konstantin Schütze[1], Gabriel G. Moya Muñoz[1,2], Nicolas D. Wendler[1,2], Douglas A. Griffith[1], Jan Lipfert[4,5*] & Thorben Cordes[1,2*]

[1] Physical and Synthetic Biology, Faculty of Biology, Ludwig-Maximilians-Universität München, Großhadernerstr. 2-4, 82152 Planegg-Martinsried, Germany

[2] Biophysical Chemistry, Department of Chemistry and Chemical Biology, Technische Universität Dortmund, Otto-Hahn-Str. 4a, 44227 Dortmund, Germany

[3] Klinikum rechts der Isar, Technische Universität München, Klinik und Poliklinik für Innere Medizin II, Ismaninger Str. 22, 81675 München

[4] Department of Physics and Center for NanoScience, Ludwig-Maximilians-Universität München, Amalienstr. 54, 80799 Munich, Germany

[5] Soft Condensed Matter and Biophysics, Department of Physics and Debye Institute for Nanomaterials Science, Utrecht University, Princetonplein 1, 3584 CC Utrecht, The Netherlands

*corresponding authors: gebhardt.christian@gmx.net, jan.lipfert@lmu.de, thorben.cordes@tu-dortmund.de

Table of Contents

A) Supplementary Note 1: Database parameter evaluation 2
B) Supplementary Note 2: Förster resonance energy transfer 7
C) Supplementary Figures 9
D) Supplementary Tables 7-9 21
E) References 22

A) Supplementary Note 1: Database parameter evaluation

Data preprocessing: the 104 PDB files of the database and the comparison PDB files were downloaded from the protein databank and preprocessed to unify the data structures. Therefore, all hetero atom entries (HETATM), anisotropy entries (ANISOU), and connection entries (CONNECT, as well as all the meta-information (REMARK) were removed from the pdb files[1,2]. Chains of polymeric protein assemblies in crystals were deleted if these were bare crystallization artifacts and do not occur in natural environments. The conservation score was calculated for all 112 chains containing the labeled residues (and the reference database) with the default settings (see Supplementary Table 1)[3,4]. Failed conservation score calculations (e.g. if too few homologue structures are available) were ignored for further analysis.

PDB data processing: The pdb files are parsed and processed with the “Bio.PDB” module[5] of the “biopython” package[6].

Parameter extraction: 28 parameters were calculated or extracted from third party software and assigned to the four categories (i) solvent exposure, (ii) residue conservation, (iii) cysteine resemblance, and (iv) secondary structure (see Supplementary Table 1-4).

Overall, 43357 and 29898 residues from the database and reference dataset are considered in the calculations, respectively. Failed parameter calculations were ignored for further analysis. Therefore, the number of calculated values varies for the 28 parameters (failure rate <10% for all parameters in the database and reference database; see Supplementary Table 5 for settings of the ConSurf-server and Supplementary Table 6 for exact numbers).

Supplementary Table 1. Solvent exposure related values were extracted using the third party algorithms (i) "Define Secondary Structure of Proteins" (DSSP) to calculate relative surface accessibility[7], (ii) "Half-Sphere-Exposure" (HSE) to calculate the number of C-alpha atoms in the half-spheres defined by the C-alpha – C-beta vector[8], and (iii) "Michel Sanner's Molecular Surface" (MSMS) to calculate the protein surface and the residue depth of the atoms[9].

#	Parameter name	Library / webserver	Extracted value	Data type
1	N_SE1_RSA_Wilke	DSSP[7]	Relative surface area with amino acid surface areas according to Wilke[10]	float
2	N_SE2_RSA_Sander	DSSP[7]	Relative surface area with amino acid surface areas according to Sander[11]	float
3	N_SE3_RSA_Miller	DSSP[7]	Relative surface area with amino acid surface areas according to Miller[12]	float
4	I_SE4_HSE1_10A	Bio.PDB[5], HSE[8]	Number of atoms in half-sphere 1 within 10 Å	integer
5	I_SE5_HSE2_10A	Bio.PDB[5], HSE[8]	Number of atoms in half-sphere 2 within 10 Å	integer
6	I_SE7_HSE1_13A	Bio.PDB[5], HSE[8]	Number of atoms in half-sphere 1 within 13 Å	integer
7	I_SE8_HSE2_13A	Bio.PDB[5], HSE[8]	Number of atoms in half-sphere 2 within 13 Å	integer
8	I_SE10_HSE1_16A	Bio.PDB[5], HSE[8]	Number of atoms in half-sphere 1 within 16 Å	integer
9	I_SE11_HSE2_16A	Bio.PDB[5], HSE[8]	Number of atoms in half-sphere 2 within 16 Å	integer
10	N_SE13_CB_SURFACE_DIST	Bio.PDB[5], MSMS[9]	Distance of the C-beta atom to the protein surface	float
11	N_SE14_MEAN_SURFACE_DIST	Bio.PDB[5], MSMS[9]	Mean distance of all atoms to the protein surface	float

Supplementary Table 2. Parameters related to residue conservation are extracted from the grades-file of the consurf server[3,4]. Settings for the ConSurf analysis are listed in Table 5.

#	Parameter name	Library / webserver	Extracted value	Data type
12	I_CS1_Color	ConSurf[3,4]	Color representation of conservation score (binned conservation score with upper and lower boundary)	integer
13	N_CS2_Score	ConSurf[3,4]	Conservation score	float
14	N_CS3_Lower_Score	ConSurf[3,4]	Lower value of confidence interval of conservation score	float
15	N_CS4_Upper_Score	ConSurf[3,4]	Upper value of confidence interval of conservation score	float
16	I_CS5_Variety_Length	ConSurf[3,4]	Number of different amino acids in homologues	integer
17	C_CS6_Cys_In_Variety	ConSurf[3,4]	Boolean: true if cysteine is in amino acid list of homologues; false else	categorical

Supplementary Table 3. Secondary structure related values were extracted using the third party algorithms “Define Secondary Structure of Proteins” (DSSP) to calculate the secondary structure of the residue of interest and its adjacent residues as well as the backbone torsion angles[7].

#	Parameter name	Library / webserver	Extracted value	Data type
18	C_SS1_SS	DSSP[7]	Secondary structure	categorical
19	N_SS2_Phi	DSSP[7]	Backbone torsion angle (n-1)-n	float
20	N_SS3_Psi	DSSP[7]	Backbone torsion angle n-(n+1)	float
21	C_SS4_SS-1	DSSP[7]	Secondary structure of predecessor residue	categorical
22	C_SS4_SS-2	DSSP[7]	Secondary structure two positions before	categorical
23	C_SS4_SS+1	DSSP[7]	Secondary structure of successor residue	categorical
24	C_SS4_SS+2	DSSP[7]	Secondary structure two positions after	categorical

Supplementary Table 4. Cysteine resemblance related values were taken from the amino acids structures to either compare the individual amino acids or group the amino acids by charge and size/mass.

**#**	**Parameter name**	**Library / webserver**	**Extracted value**	**Data type**
25	C_CR1_Name	-	Amino acid (name)	categorical
26	N_CR2_Mass	-	Mass of amino acid [u]	float
27	C_CR3_Charge	-	Charge of amino acid in buffer solution at pH=7.4 [e]	categorical
28	I_CR4_N_Sidechain	-	Number of sidechain atoms (without H-atoms)	integer

Supplementary Table 5. **ConSurf-server settings.** Overview of all user parameters set for the conservations score calculation on https://consurf.tau.ac.il/ (accessed January 24th, 2021).

**Parameter**	***Value***
DNA_AA	AA
NMR	no
PDB_yes_no	yes
MSA_yes_no	no
Homolog_search_algorithm	HMMER
ITERATIONS	1
E_VALUE	0.0001
proteins_DB	UNIREF90
user_select_seq	no
MAX_NUM_HOMOL	150
best_uniform_sequences	uniform
MAX_REDUNDANCY	95
MIN_IDENTITY	35
MSAprogram	MAFFT
ALGORITHM	Bayes
SUB_MATRIX	BEST

Supplementary Table 6. **Parameter overview and statistics.** The table summarizes the number of analyzed residues (complete database, labeled residues) and the statistical evaluation of the parameter scores with respect to mean-square deviation (MSD), gini coefficient, and adapted Shannon entropy (see methods for details).

#	Name	Number of all analyzed residues	Number of labeled residues	MSD	Gini coeff	Shannon entropy
1	N_SE1_RSA_Wilke	40056	385	0.951	0.303	0.931
2	N_SE2_RSA_Sander	40056	385	0.866	0.323	0.923
3	N_SE3_RSA_Miller	40056	385	0.948	0.330	0.922
4	I_SE4_HSE1_10A	40056	385	0.674	0.663	0.687
5	I_SE5_HSE2_10A	40056	385	0.259	0.421	0.851
6	I_SE6_HSE1_13A	40056	385	0.922	0.625	0.712
7	I_SE7_HSE2_13A	40056	385	0.266	0.385	0.861
8	I_SE8_HSE1_16A	40056	385	0.972	0.596	0.744
9	I_SE9_HSE2_16A	40056	385	0.206	0.347	0.895
10	N_SE10_CB_SURFACE_DIST	40056	385	0.210	0.585	0.721
11	N_SE11_MEAN_SURFACE_DIST	40056	385	0.883	0.578	0.744
12	I_CS1_Color	39409	376	0.322	0.271	0.940
13	N_CS2_Score	39409	376	0.856	0.339	0.914
14	N_CS3_Lower_Score	39409	376	0.869	0.469	0.819
15	N_CS4_Upper_Score	39409	376	0.530	0.343	0.900
16	I_CS5_Variety_Length	39409	376	0.677	0.354	0.911
17	C_CS6_Cys_In_Variety	39409	376	0.006	0.038	0.996
18	C_SS1_SS	41502	390	0.218	0.307	0.894
19	N_SS2_Phi	41502	390	0.448	0.370	0.869
20	N_SS3_Psi	41502	390	0.231	0.339	0.883
21	C_SS4_SS-1	41227	385	0.258	0.383	0.861
22	C_SS5_SS-2	41018	382	0.175	0.250	0.919
23	C_SS6_SS+1	41227	388	0.218	0.316	0.900
24	C_SS7_SS+2	41021	385	0.207	0.259	0.917
25	C_CR1_Name	43357	396	0.753	0.401	0.909
26	N_CR2_Mass	43357	396	0.361	0.367	0.905
27	C_CR3_Charge	43357	396	0.156	0.162	0.956
28	I_CR4_N_Sidechain	43357	396	0.413	0.368	0.902

B) Supplementary Note 2: Förster resonance energy transfer

Förster radius calculation:

Spectral information, quantum yield, and extinction coefficients are taken from the database https://www.fpbase.org/spectra/ and provided with the labelizer-package for the most commonly used fluorophores.

The Förster radius R_0 is given by

$$R_0{}^6 = \frac{9\,ln\,(10)}{128\,\pi^5\,N_A}\frac{\kappa^2}{n^4}Q_D\,\frac{\int_0^\infty F_D(\lambda)\varepsilon_{A_{max}}\varepsilon_A(\lambda)\,\lambda^4\,d\lambda}{\int_0^\infty F_D(\lambda)\,d\lambda}, \tag{1}$$

whereby Q_D is the donor quantum yield, F_D the normalized donor emission spectrum, ε_A the normalized acceptor emission spectrum, and $\varepsilon_{A_{max}}$ the acceptor extinction coefficient.

The following values are set fix to theoretical values:

Orientation factor κ^2:	2/3
Averaged refractive index n (ref. [13]):	1.4

Distance screening:

Center of mass of a sphere with radius R cut with a cone of angle α in the z-dimension (spherical sector):

$$\vec{d} = \frac{\int_0^{\pi-\alpha} d\theta \int_0^{2\pi} d\phi \int_0^R dr\;r^2\sin(\theta)\cdot\vec{r}}{\int_0^{\pi-\alpha} d\theta \int_0^{2\pi} d\phi \int_0^R dr\;r^2\sin(\theta)\cdot 1} = \begin{pmatrix}0\\0\\1\end{pmatrix}\cdot\frac{3}{8}R(1-\cos(\alpha))$$

We assume the origin to be at the attachment site (C-ß atom) of the fluorophore and model the accessible volume of the dye with a cut sphere of angle α and the non-accessible space with $\pi-\alpha$. We approximate the center of mass of the non-accessible volume $\vec{d}'$ with the center of mass of all N atom positions $\vec{r_i}$ within the protein closer than R to the attachment point (see Figure 5):

$$\vec{d}' = \frac{1}{N}\sum_i \vec{r_i}\;\forall\, i\; with\; |\vec{r_i} < R|$$

The direct relation between the center of mass of atoms in the protein $\vec{d}'$ and the center of mass of the fluorophores accessible-volume $\vec{d}$ is given by:

$$\vec{d} = \left(\frac{\vec{d}'}{R} - \frac{3}{4}\frac{\vec{d}'}{|\vec{d}'|}\right)R$$

We add an empirically determined correction factor based on simulations with 35 different fluorophore parameters on 100 residue pairs in 10 different protein structures to account for the finite size of atoms and fluorophores, which leads to a gap between protein atoms and accessible volume (see Supplementary Figure 7B/D).

The offset for the protein surface is added as a small addition to the fluorophore linker

$$\vec{d} = \left(\frac{\vec{d}'}{R} - \frac{3}{4}\frac{\vec{d}'}{|\vec{d}'|}\right)(R + \varepsilon)$$

and reads as

$$\varepsilon = \max(R_A, 2\min(R_1, R_2, R_3) - R_A) + 0.014 * R - 0.0059 * R^2 \,.$$

This correction can reproduce the simulated mean positions $\vec{R}_{MP}$ with a root mean square deviation of ±2.7 Å (see Supplementary Figure 7F) and mean position distances $R_{MP} = |\vec{R}_{MP,1} - \vec{R}_{MP,2}|$ with a root mean square deviation of ±2.1 Å (see Figure 8C).

We approximated the relation between the mean positions of the accessible volumes to the measured FRET averaged distances as

$$R_{<E>} = R_{MP} + A\, e^{-b\, R_{MP}} \,,$$

whereby the second term accounts for FRET-efficiency weighted averaging effects at small distances (see Supplementary Figure 8B). The values A and b are determined as $A = 20.6$ Å and $b = 0.037$ $1/$Å from a fit to the 35000 simulated distances within the ten selected protein structures, which is similar to the reported relations in ref. [14] and [15] for DNA. With this relationship, the simulated distance with FPS is reproduced up to a deviation of ±3.4 Å (±3.1 Å for distances between 40 and 75 Å).

Based on the corrected FRET values, the (screening) FRET-efficiency of a residue pair {i,j} is calculated as

$$E_{i,j} = \frac{1}{1 + \left(\frac{R_{<E>}}{R_0}\right)^6} \,.$$

<u>Distance refinement</u>

A refinement is calculated based on the N highest FRET scores (with default N=300) using the available FPS simulation software[16] with standard parameter settings (see Supplementary Table 8).

C) Supplementary Figures

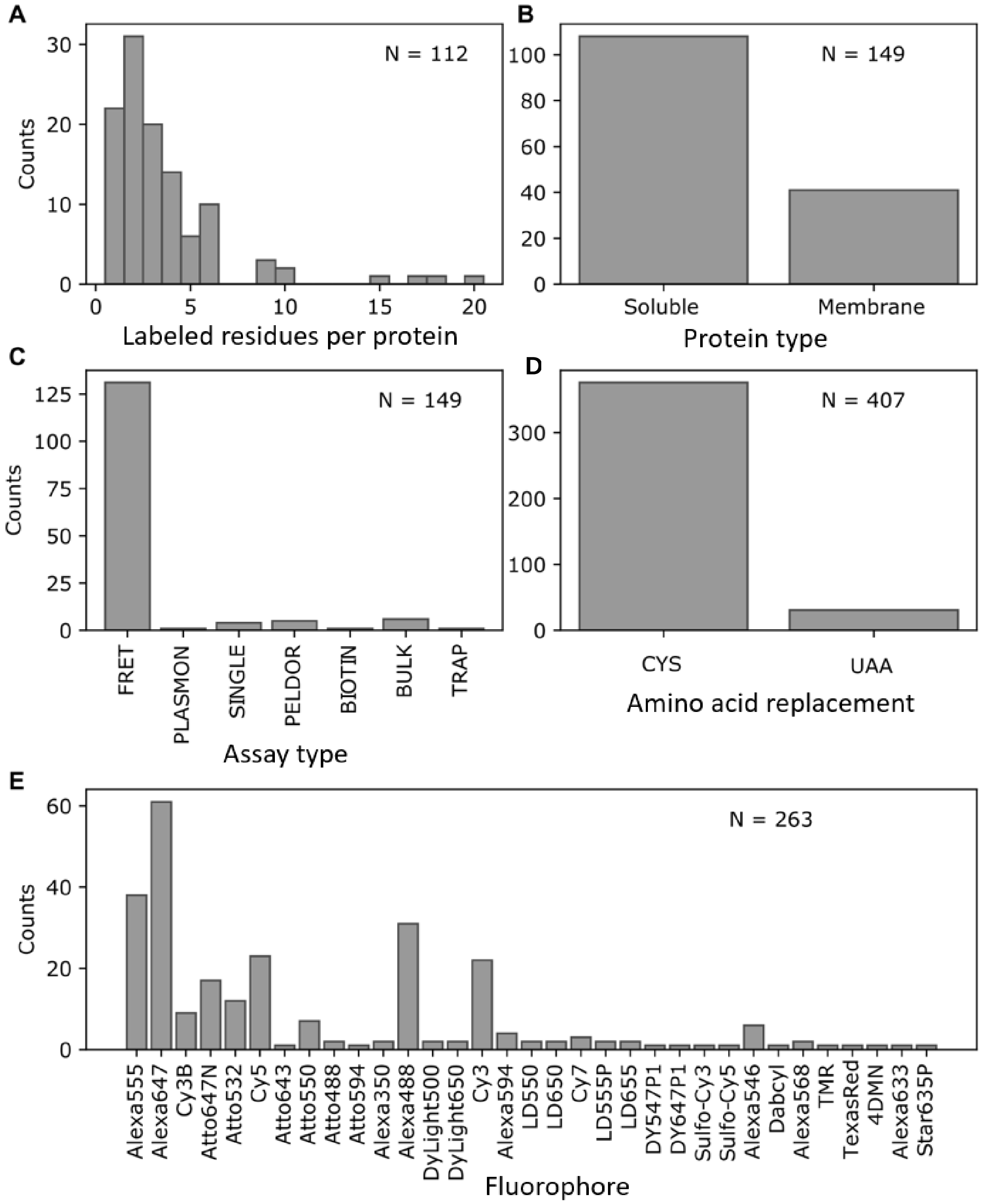

Supplementary Figure 1. Labeling database statistics. A) Number of labeled residues per chain in the database with N=112 different protein chains. **B)** Comparison of published protein systems with soluble and membrane proteins (N=149 published protein systems, multiple occurrence possible). **C)** Statistics of the different assay types used for the labeling database (N=149 published protein systems, multiple occurrence possible). Around 90% of the assays are single-molecule FRET assays (FRET), the others are bulk FRET (BULK) or single fluorophore labeled (SINGLE) assays, spin labels (PELDOR), gold labels (PLASMON), biotin labels (BIOTIN) and linker labels for optical traps (TRAP). **D)** Statistics on the labeling residues (cysteine or unnatural amino acid, N=407 residues). **E)** Statistics on the fluorophores used in the publications (N=263 occurrences in the publications).

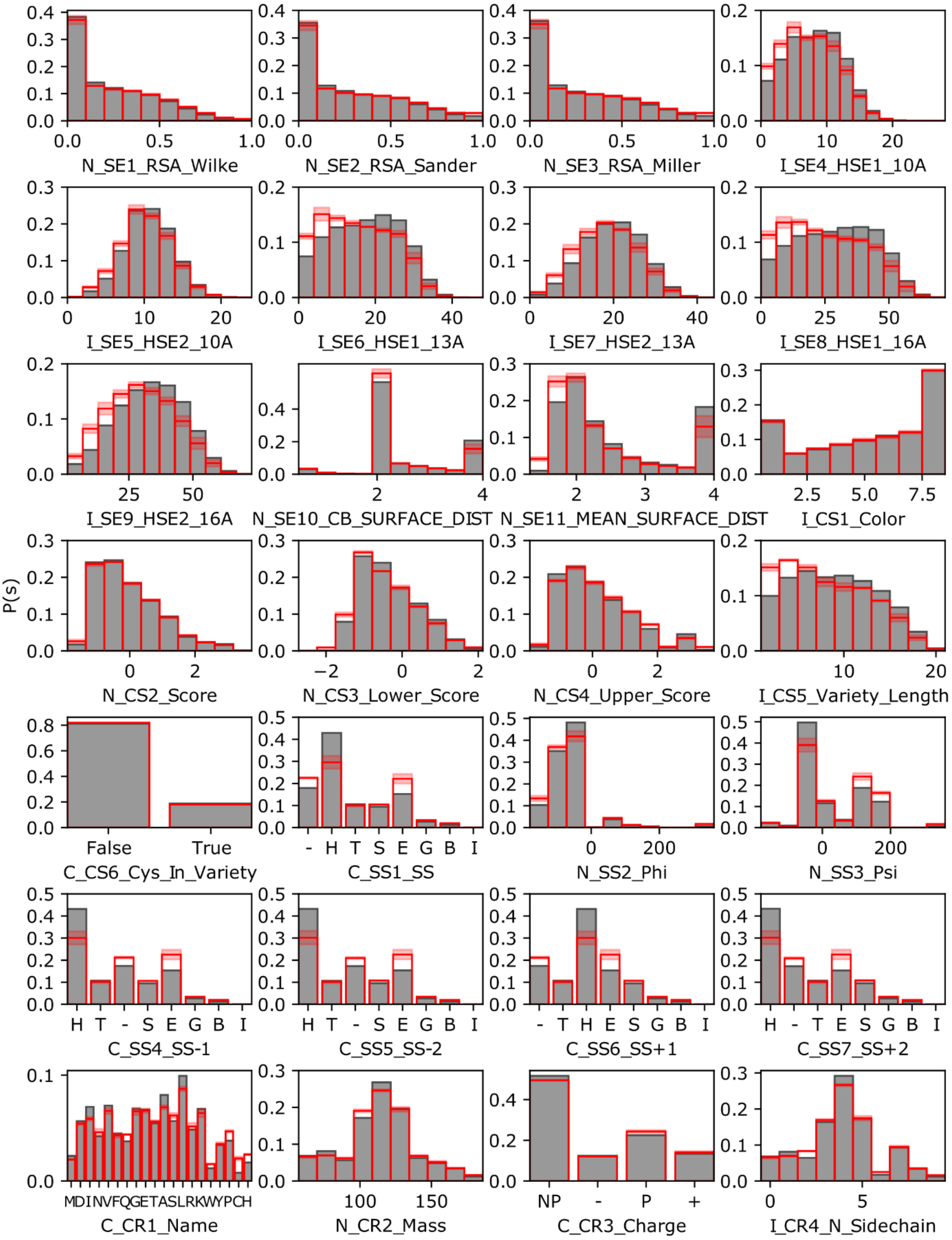

Supplementary Figure 2. Parameter distribution comparison. Probability distributions $P(s)$ of all 28 scores in the labeling database (gray). The values are compared to a randomly selected representative reference dataset (red line: mean values, pale area: standard deviation of triplicates) based on the pdbselect dataset[17,18] (see methods for details). The x-axis label specifiers the parameter: first part for the type of data (I: integer, N: numeric, C: categorical), second part for the parameter group (SE: solvent exposure, CS: conservation score, SS: secondary structure, CR: cysteine resemblance), and the rest for a reasonable name (see methods for details).

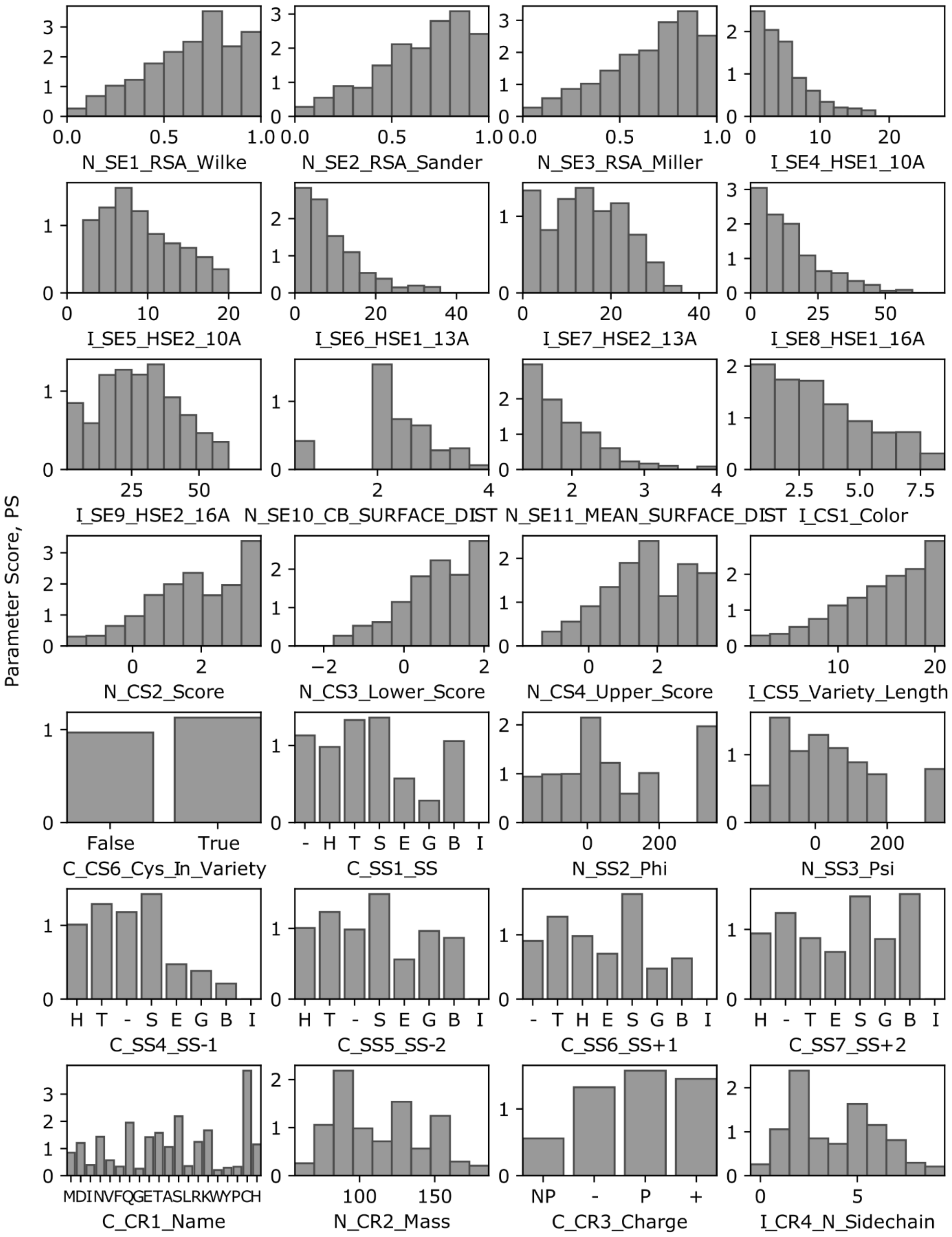

Supplementary Figure 3. Conditional frequencies of parameters. Conditional frequency distributions $P(s|l)/P(s)$ defining the parameter scores of all 28 parameters in the labeling database. The x-axis label specifiers the parameter: first part for the type of data (I: integer, N: numeric, C: categorical), second part for the parameter group (SE: solvent exposure, CS: conservation score, SS: secondary structure, CR: cysteine resemblance), and the rest for a reasonable name (see methods for details).

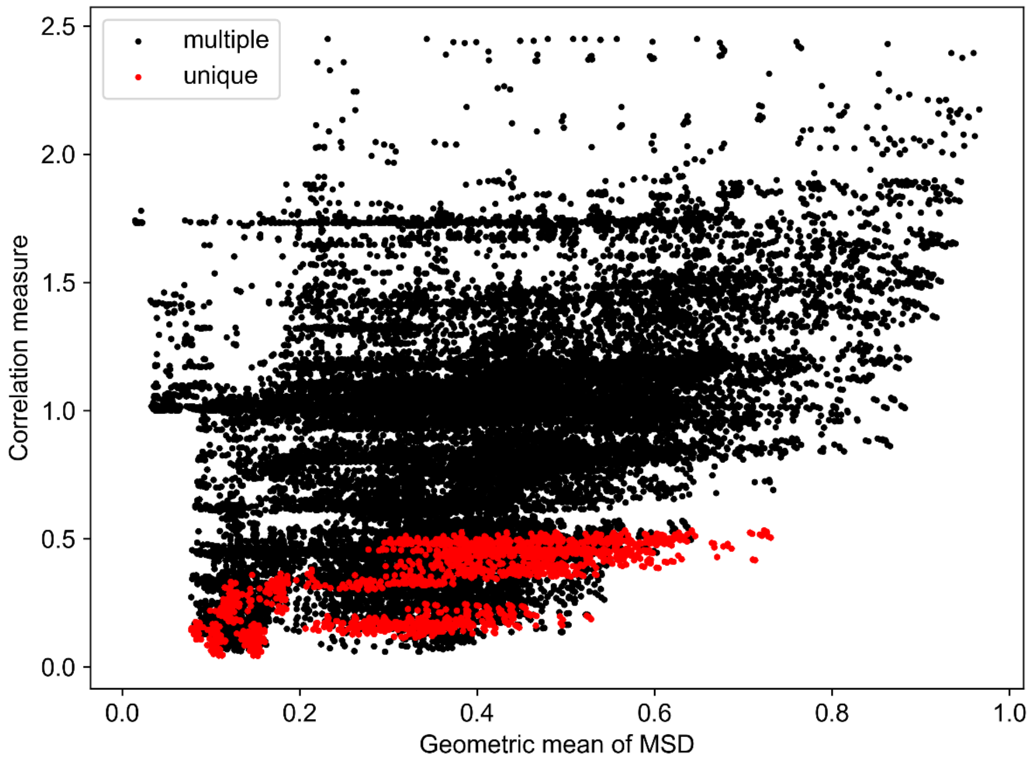

Supplementary Figure 4. Correlation measure and averaged mean square deviation (MSD).
The plot shows the geometric mean of the MSD value of the selected parameters (mean square deviation from equal contribution, see methods) versus the correlation measure (2-norm of all correlations). All parameters are combined with each other to sets of 4, whereby points with multiple (2 or more) parameters from the same group (e.g. solvent exposure) are marked black. Combinations with one parameter from each group are marked red.

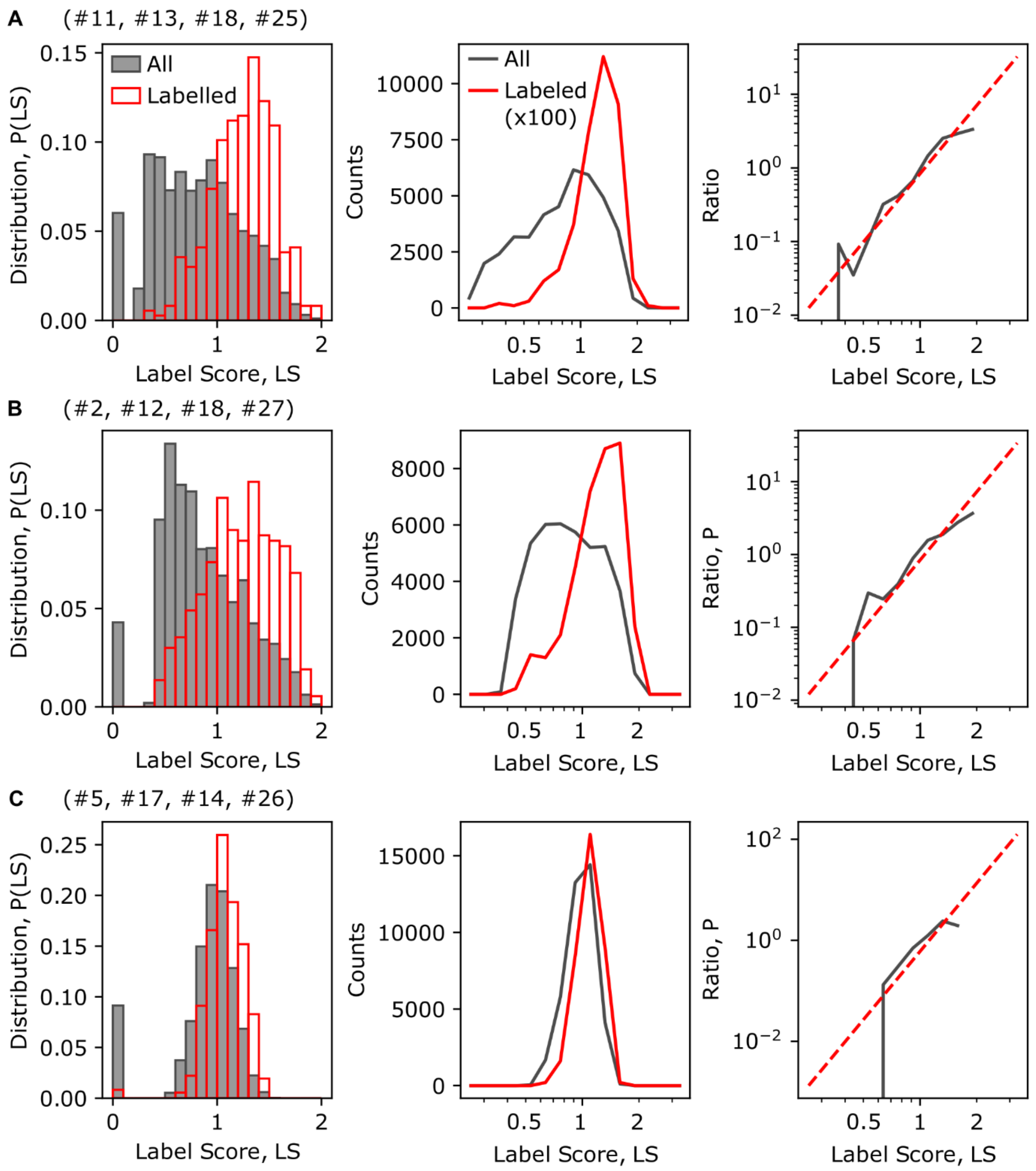

Supplementary Figure 5. Label score evaluation for different parameter sets. A Label score probability distribution of all residues (gray) and labeled residues (red) in our database (left) and the a histogram with logarithmic scale of the label scores (middle) for the selected quadruple (#11: mean surface distance, #13: ConSurf score, #18: secondary structure, #25: amino acid identity) (default settings). The ratio of the probability distribution of labeled and all residues (gray) is fitted with a linear dependency (red, dashed) in the log-log-plot (right). **B** Same evaluation as in A for another suitable parameter selection of the quadruple (#2: relative surface area Sander, #12: ConSurf color, #18: secondary structure, #27: amino acid charge). **C** Same evaluation as in A for a parameter set with poor prediction power (#5: second half of 10 Å half-sphere exposure, #17: cysteine in homologue structures, #14: secondary structure two positions after, #26: amino acid mass).

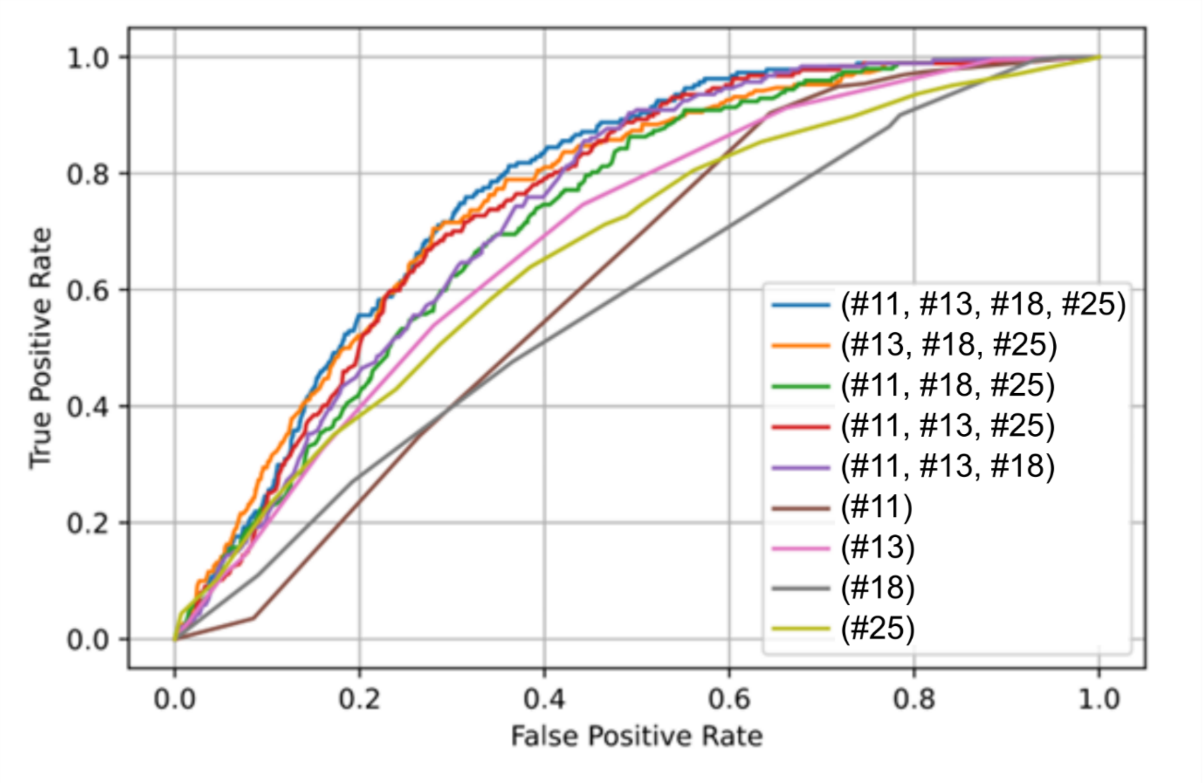

Supplementary Figure 6: ROC curves for reduced parameter sets. To study the importance of individual parameters in the final prediction, we compared the receiver operating characteristic (ROC curve) for the baseline, when removing one of four parameters and for each parameters on its own. We considered labeled sites positives and unlabeled site negatives, which most likely overestimates the false-positive rate.

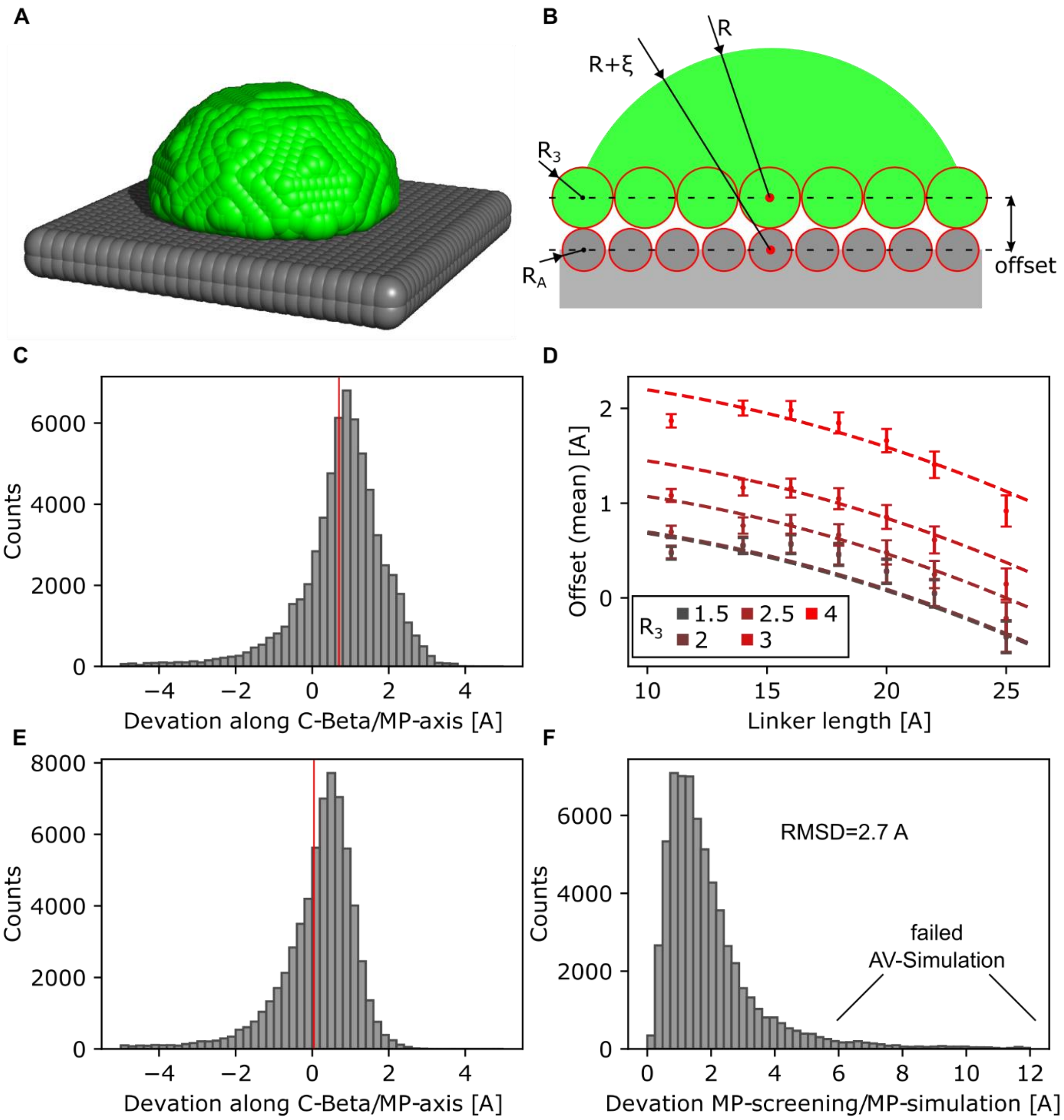

Supplementary Figure 7. Correction parameter for mean position of accessible volume. (A) Simplified accessible volume simulation (green) in an idealized system of a planar array of atoms (gray). **(B)** Motivation for the correction factor is illustrated with the offset between the atom coordinates (lower dashed line) and the accessible volume coordinates (upper dashed line). The correction factor ε corrects for this gap between accessible surface (green) and inaccessible surface (gray) under the consideration of the linker length (R, corresponds to the AV radius), the atom radius R_A and the smallest fluorophore radius R_3 of the ellipsoidal approximation[14,16,19]. **(C)** Deviation between simulated mean position of accessible volume (FPS software) and estimated mean position (SSM approach) with indicated mean value (red line). **(D)** Mean offset from (C) for different linker lengths R and fluorophore radii R_3 is shown with error bars (standard error of the mean from simulations). The estimation of the offset in (C) is fitted globally with the correction factor $\varepsilon = \max(R_A, 2\min(R_1, R_2, R_3) - R_A) + 0.014 * R - 0.0059 * R^2$ (dashed lines). **(E)** Deviation between simulated mean position of accessible volume (FPS software) and estimated mean position (SSM approach) including the correction factor ε (mean value: red line). **(F)** Distance between corrected screening mean position (SSM approach) and simulated mean position (FPS software) results in a deviation of 2.7 Å (RMSD). The large deviations for some positions (>6 Å) result from failed FPS simulations (unreasonable small accessible volumes due to interfering atoms close to the linker attachment site).

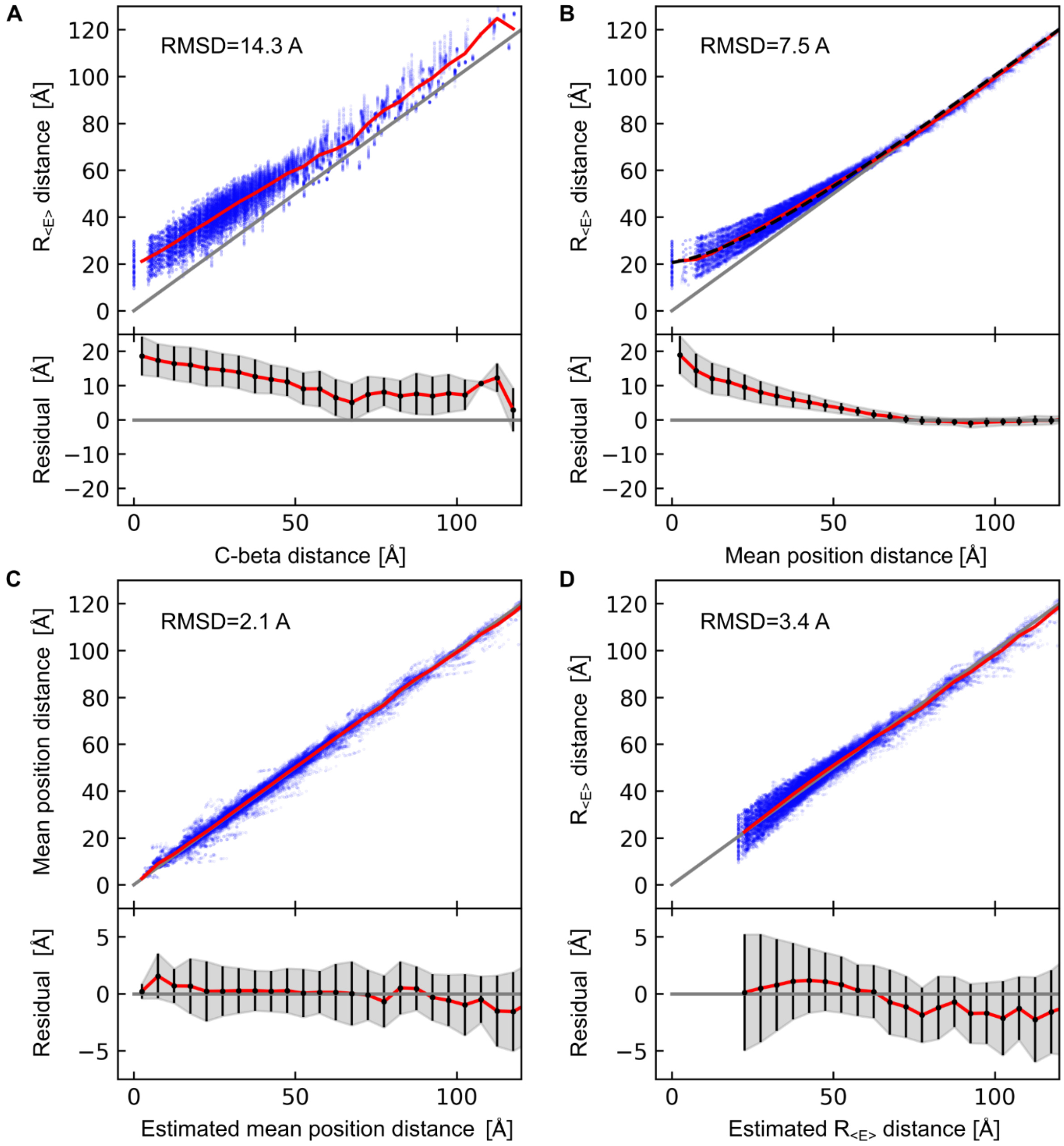

Supplementary Figure 8. Correction parameter for distance simulation. (A) C-beta distances are plotted against simulated distances with FPS-software (blue datapoints) with mean values (red line). The bottom axis shows the mean residual (red line) and the standard deviation interval (error bars / gray area) on binned data from the top. **(B)** Mean dye position distances R_{MP} (center of mass of the AV-simulation) are plotted against the FRET-averaged distances (blue datapoints) with mean values (red line). The mean values are fitted to the curve $R_{MP} + A\ e^{-b\ R_{MP}}$ with $A = 20.6$ Å and $b = 0.037$ 1/Å (black dashed line). **(C)** Mean dye position estimations based on the spherical sector calculation (SSM) approach are plotted against mean dye position distances R_{MP} from FPS-simulation software. **(D)** Mean dye position distances from the SSM-estimation are converted to FRET-averaged distances with the correction factors from (B) and plotted against the simulated $R_{<E>}^{model}$ from FPS-simulation.

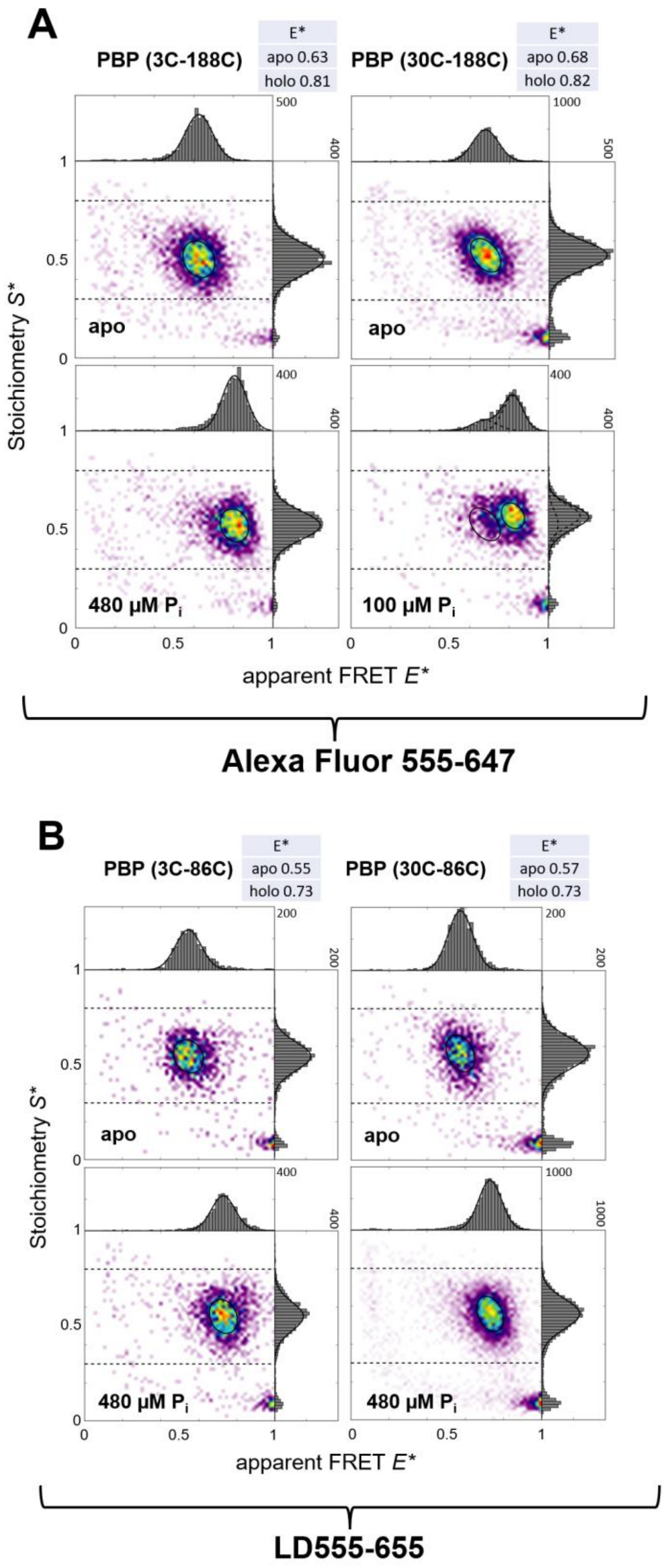

Supplementary Figure 9. smFRET Characterization of four distinct PBP double-cysteine variants with two different fluorophore pairs with similar Förster radius. ALEX histograms with 61 bins of apo and holo states of PBP variants as indicated labelled with **(A)** Alexa Fluor 555-647 and **(B)** LD555-655 dyes. Mean values for E* are background corrected apparent FRET efficiencies analyzed by a dual-colour burst search with additional per-bin thresholds of all photons >150.

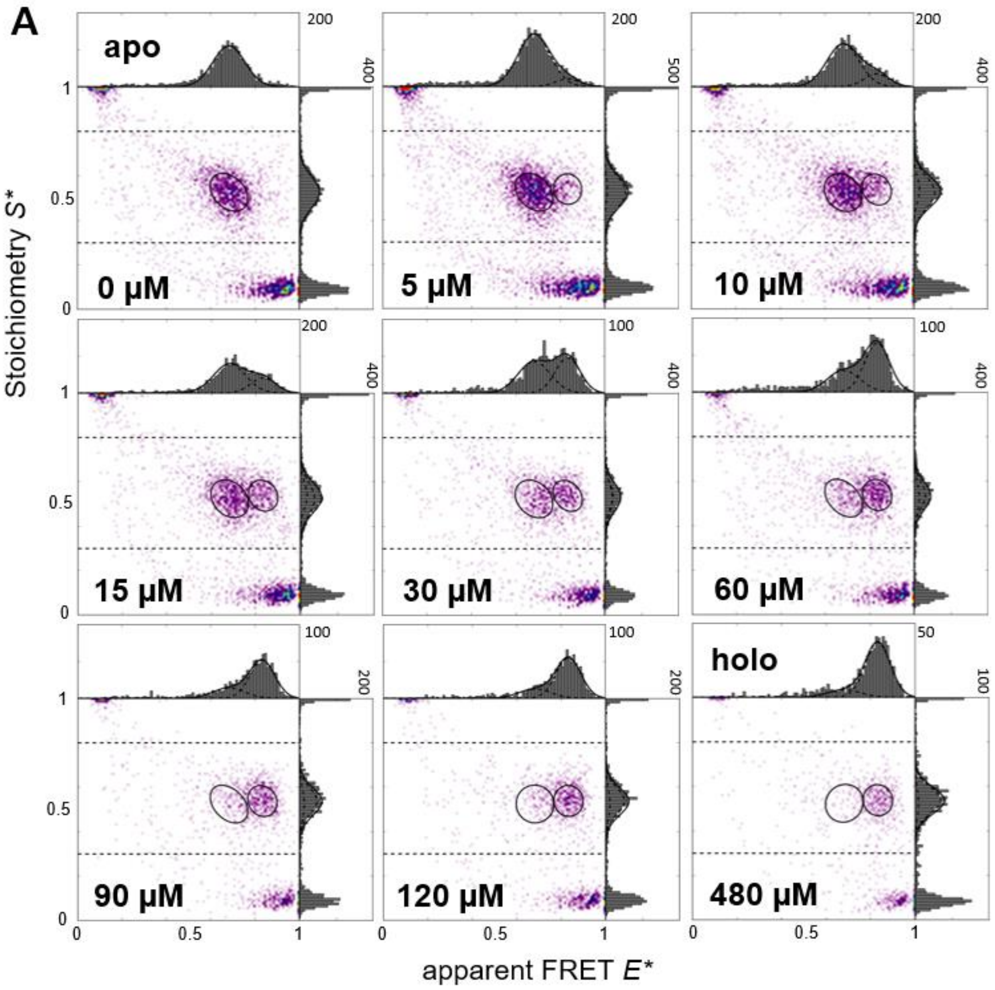

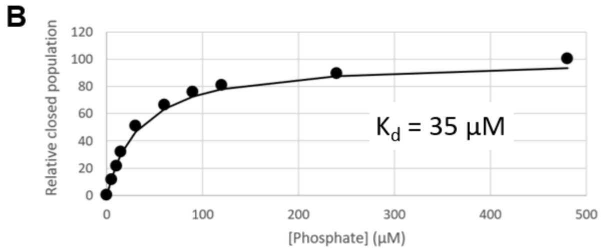

C

PBP variant (n = 2)	K_d (labelled)
PBP (3/188)	26±7 μM
PBP (30/188)	30±5 μM
PBP (3/86)	16±6 μM
PBP (30/86)	27±17 μM

Supplementary Figure 10. Biochemical characterization of labelled PBP variants using smFRET. (A) Representative data of S3-188C labelled with Alexa Fluor 555-647 at indicated phosphate concentrations including a two-state fit of low FRET apo and high-FRET holo state. **(B)** The binding curve was calculated from smFRET measurements considering the closed fraction (r_{closed}) as a function of the substrate concentration. **(C)** Determined mean Kd-values for all four labelled PBP variants including standard deviation.

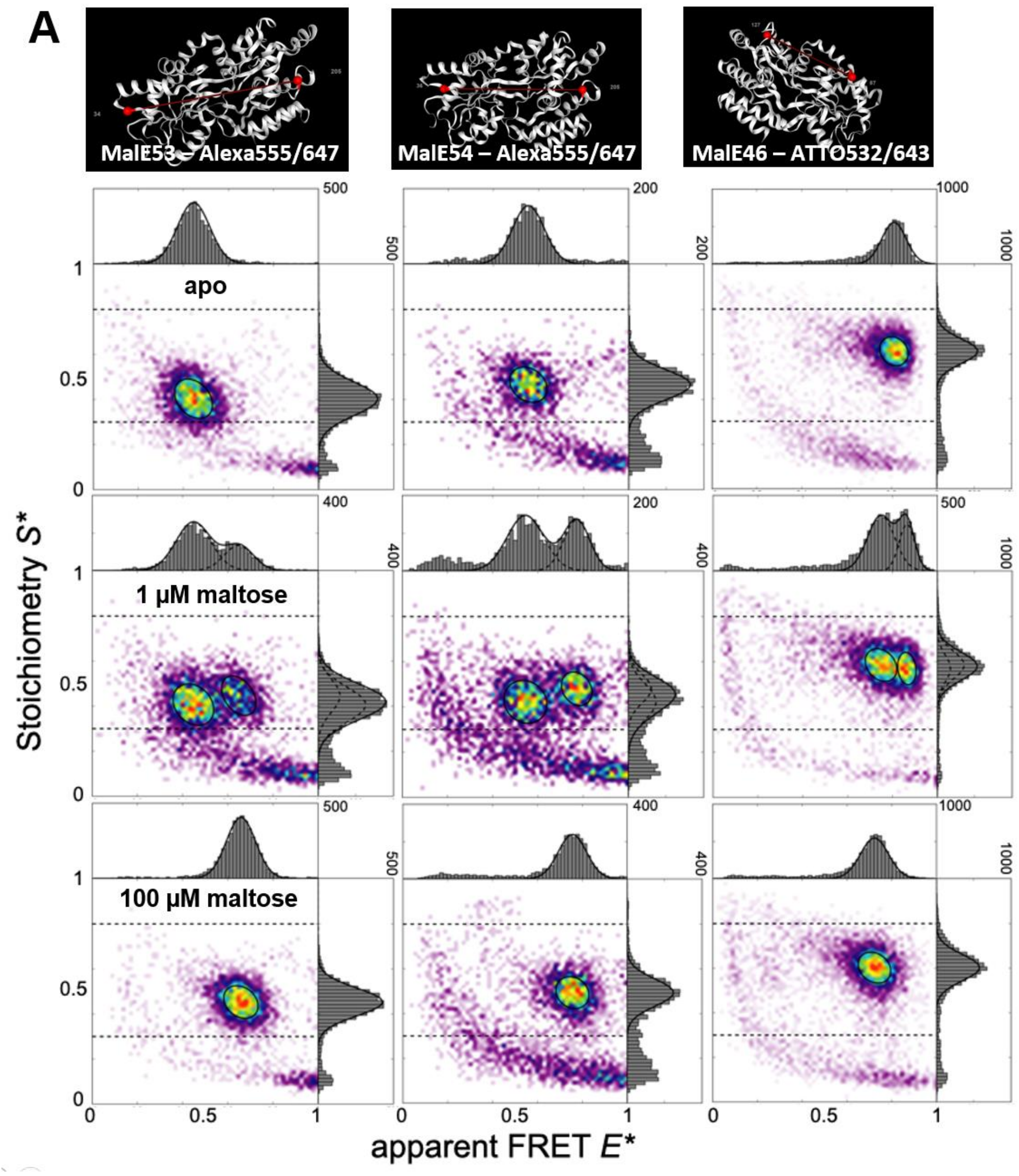

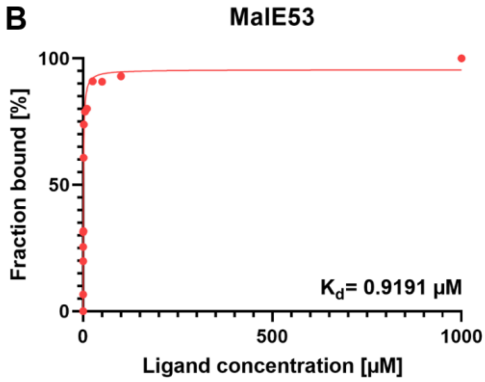

C

MalE variant (n = 3)	K_d (labelled)
MalE53 (34/205)	1.3±0.6 µM
MalE54 (36/205)	1.1±0.2 µM
MalE46 (87/127)	0.9±0.2 µM

D

Dye pair	R_0 (theo)	R_0 (exp)
Alexa555-647	4.93 nm	5.1 nm
Alexa546-647	6.86 nm	6.5 nm
ATTO532-643	5.75 nm	5.8 nm

Supplementary Figure 11. New smFRET and biochemical data of MalE variants used in accurate FRET analysis. (A) Label positions and smFRET of the respective variant for ligand-free apo, 1 µM maltose and saturated maltose (100 µM, holo). **(B)** Representative results from affinity titrations of MalE 53 with Alexa555-647 and **(C)** mean and standard deviation of determined Kd-values for all three labelled variants.

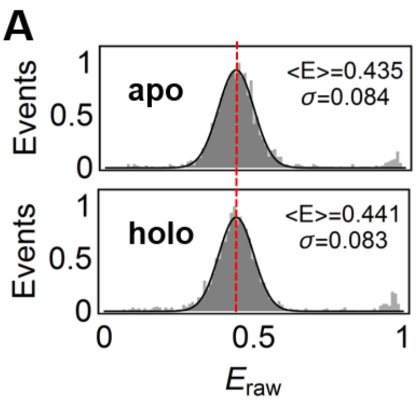

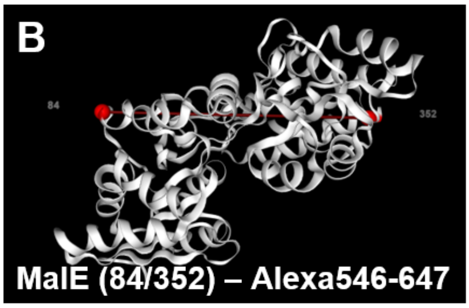

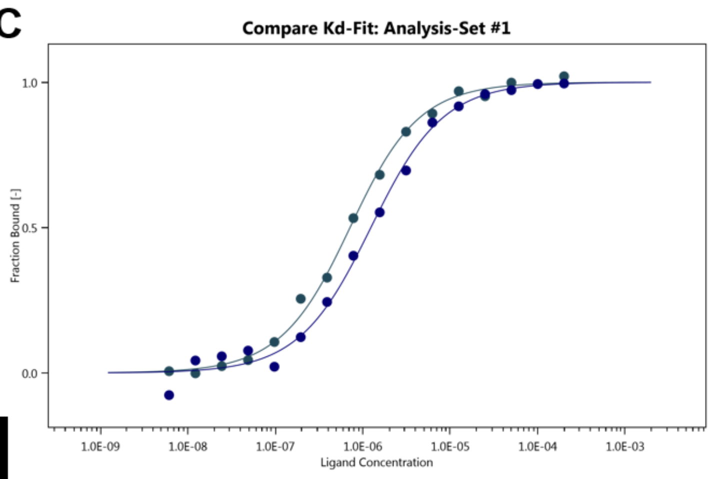

D

Correction parameters			
α = 0.052	β = 1.65	δ = 0.082	γ = 0.44

E

MalE state	E_{ac} (exp)	E_{ac} (theo)	d (exp)	d (theo)
Apo (FS = 1.32)	0.53	0.51	6.37 nm	6.44 nm
Holo (FS = 1.13)	0.55	0.53	6.29 nm	6.38 nm

Supplementary Figure 12. Biophysical and biochemical characterization of MalE variant with optimized FRET efficiency values but minimal FRET changes between apo and holo state. (A) Unccorrected FRET efficiency histogram of **(B)** MalE (84/352) labeled with Alexa Fluor 546-647 where ligand binding was verified by label-free microscale thermophoresis **(C)**. Conversion of the data into accurate FRET efficiency values was done using additional data with distinct FRET efficiency values (yet use of the same fluorophore pair on MalE) and resulted in correction parameters **(D)** and accurate FRET efficiency values and distances using a Förster radius of 6.5 nm **(E)**.

D) Supplementary Tables 7-9

Supplementary Table 7. **Parameter analysis.** We compared the baseline model, the model with one of the four parameters removed and the predictive power of each parameter on its own. We show the difference between the means and the T value when comparing the distributions of P(s) and P(s|l), the average difference between the means after 400x bootstrapping together with the 95% CI intervals. The difference of means in our bayesian model is a metric for the model's ability to separate the labeled residues from the background. Note that the median derived from bootstrapping of the differences of the distribution means diverges from the main analysis.

**Parameters**	**Difference of P(s|l) and P(s) means**	**T value**	**400x bootstrapping median of the difference of P(s|l) and P(s) means [95% confidence interval]**
#11, #13, #18, #25	0.45	19.23	0.41 [0.38-0.47]
#13, #18, #25	0.33	18.19	0.27 [0.24-0.34]
#11, #18, #25	0.30	15.88	0.31 [0.28-0.33]
#11, #13, #25	0.40	18.01	0.34 [0.30-0.42]
#11, #13, #18	0.36	19.67	0.32 [0.29-0.39]
#11	0.13	12.91	0.14 [0.12-0.17]
#13	0.18	16.98	0.12 [0.09-0.19]
#18	0.07	11.94	0.06 [0.05-0.08]
#25	0.11	8.51	0.11 [0.08-0.13]
#11, #13, #18, #25	0.45	19.23	0.41 [0.38-0.47]

Supplementary Table 8. **FPS settings.** Overview of all user parameters set for the runtime analysis of the distance refinement simulation with the FPS software[16]. The default settings for the labelizer package and webserver use discStep = 0.8 and nsamples = 100000 (all other parameters depend on the selected fluorophore pair).

LabelLib.dyeDensityAV3

**Parameter**	***Value***
discStep	0.8 (coarse-grained: 1.2)
linkerLength	varied
linkerDiameter	4.5
dyeRadii	varied

LabelLib. meanEfficiency

**Parameter**	***Value***
R0	57.5
nsamples	100000 (coarse-grained: 10000)

Supplementary Table 9. **Spherical sector vs. FPS runtime comparison.** Calculation time overview of the fast screening method (spherical sector calculation) and a coarse-grained distance refinement simulation (FPS software[16]) with 3500 distance pairs per pdb-file (100 distances, 35 different dye parameter) and a refined FPS simulation with 1400 distance pairs per pdb-file (40 distances, 35 different dye parameter).

**PDB**	**Molar mass [u]**	**SSM time per DA-pair [ms]**	**Coarse-grained FPS time per DA-pair [ms]**	**FPS time per DA-pair [ms]**
3L6G	28,830	0.81±0.04	103±5	315±11
2KHO	65,650	0.84±0.01	166±5	419±8
2CG9	188,730	0.94±0.01	303±12	647±10
4B1O	831,160	4.31±0.09	1113±36	2087±46
172L	18,730	0.67±0.01	77±2	249±10
2A65	59,750	0.93±0.04	169±5	474±9
1WDN	25,130	0.78±0.01	100±3	315±4
5XPD	33,310	0.67±0.02	94±3	282±10
1P7B	74,510	0.80±0.01	156±12	398±14
1HKA	17,970	0.81±0.04	81±2	259±11

E) Supplementary References

1. Bernstein, F. C. *et al.* The protein data bank: A computer-based archival file for macromolecular structures. *J. Mol. Biol.* **112**, 535–542 (1977).
2. wwPDB. *Protein Data Bank Contents Guide:Atomic Coordinate Entry Format DescriptionVersion 3.30*. https://www.wwpdb.org/documentation/file-format (2012).
3. Glaser, F. *et al.* ConSurf: identification of functional regions in proteins by surface mapping of phylogenetic information. *Bioinformatics* **19**, 163–164 (2003).
4. Ashkenazy, H. *et al.* ConSurf 2016: an improved methodology to estimate and visualize evolutionary conservation in macromolecules. *Nucleic Acids Res.* **44**, W344-50 (2016).
5. Hamelryck, T. & Manderick, B. PDB file parser and structure class implemented in Python. *Bioinformatics* **19**, 2308–2310 (2003).
6. Cock, P. J. A. *et al.* Biopython: freely available Python tools for computational molecular biology and bioinformatics. *Bioinformatics* **25**, 1422–1423 (2009).
7. Kabsch, W. & Sander, C. Dictionary of protein secondary structure: pattern recognition of hydrogen-bonded and geometrical features. *Biopolymers* **22**, 2577–2637 (1983).
8. Hamelryck, T. An amino acid has two sides: A new 2D measure provides a different view of solvent exposure. *Proteins Struct. Funct. Genet.* **59**, 38–48 (2005).
9. Sanner, M. F., Olson, A. J. & Spehner, J.-C. Reduced surface: An efficient way to compute molecular surfaces. *Biopolymers* **38**, 305–320 (1996).
10. Tien, M. Z., Meyer, A. G., Sydykova, D. K., Spielman, S. J. & Wilke, C. O. Maximum Allowed Solvent Accessibilites of Residues in Proteins. *PLoS One* **8**, e80635 (2013).
11. Rost, B. & Sander, C. Conservation and prediction of solvent accessibility in protein families. *Proteins Struct. Funct. Bioinforma.* **20**, 216–226 (1994).
12. Miller, S., Janin, J., Lesk, A. M. & Chothia, C. Interior and surface of monomeric proteins. *J. Mol. Biol.* **196**, 641–656 (1987).
13. Voith von Voithenberg, L. & Lamb, D. C. Single Pair Förster Resonance Energy Transfer: A Versatile Tool To Investigate Protein Conformational Dynamics. *BioEssays* **40**, 1700078 (2018).
14. Sindbert, S. *et al.* Accurate Distance Determination of Nucleic Acids via Förster Resonance Energy Transfer: Implications of Dye Linker Length and Rigidity. *J. Am. Chem. Soc.* **133**, 2463–2480 (2011).
15. Hellenkamp, B. *et al.* Precision and accuracy of single-molecule FRET measurements—a multi-laboratory benchmark study. *Nat. Methods* **15**, 669–676 (2018).
16. Kalinin, S. *et al.* A toolkit and benchmark study for FRET-restrained high-precision structural modeling. *Nat. Methods* **9**, 1218–1225 (2012).
17. Hobohm, U., Scharf, M., Schneider, R. & Sander, C. Selection of representative protein data sets. *Protein Sci.* **1**, 409–417 (1992).
18. Griep, S. & Hobohm, U. PDBselect 1992–2009 and PDBfilter-select. *Nucleic Acids Res.* **38**, D318–D319 (2010).
19. Gebhardt, C. *et al.* Molecular and Spectroscopic Characterization of Green and Red Cyanine Fluorophores from the Alexa Fluor and AF Series**. *ChemPhysChem* **22**, 1566–1583 (2021).
