# Supplementary material for: Labelizer: systematic selection of protein residues for covalent fluorophore labeling
Source: Nat Commun. 2025 May 4;16:4147. doi: 10.1038/s41467-025-58602-y (PMC12049551; doi:10.1038/s41467-025-58602-y)
Supplement: Supplementary file 4 — Description of Additional Supplementary Files [file 41467_2025_58602_MOESM4_ESM.pdf]

## Description of Additional Supplementary files

### Supplementary Data

Description: The supplementary data Excel file provides various data and information related to this manuscript. The first tab is the used database in form of pdb entries of successfully labelled proteins and their related references. The second and third tabs list the label score values for the two test proteins of the study: PBP and MalE. The fourth and fifth tabs provide the correlation of the degree of labelling with the label score and biochemical data for the random MalE variants. The final tab is the overview of data related to accurate FRET analysis and the correlation of FRET score with experimental and theoretical outputs.
